# Current Concepts in Radial Club Hand

**DOI:** 10.2174/1874325001711010369

**Published:** 2017-04-28

**Authors:** Takehiko Takagi, Atsuhito Seki, Shinichiro Takayama, Masahiko Watanabe

**Affiliations:** 1 Department of Orthopaedic Surgery, Surgical Science, Tokai University School of Medicine, Isehara, Kanagawa, Japan; 2 Department of Orthopaedic Surgery, National Center for Child Health and Development, Setagaya, Tokyo, Japan

**Keywords:** Centralization, Radialization, Radial club hand, Radius lengthening

## Abstract

Radial club hand is a complex congenital abnormality of the radial or pre-axial border of the upper extremity. It has a wide range of phenotypes from hypoplasia of the thumb to complete absence of the radius and the first ray. Centralization with tendon transfer is a popular method for maintaining the correct position of radial club hand. On the other hand, various corrections were devised, *e.g*. radialization after distraction to emphasize the fact that the head of the ulna is positioned under the radial carpal bones and is no longer placed in a slot in the center of the carpus, microvascular epiphysis transfer, gradual correction using Ilizarov method, for Bayne Type III or Type IV. We should pay attention to the recurrence of radial deformity or circulatory impairment with the tension. Lunate excision or ulnar shortening can be selected for tension-free correction. Radialization can be indicated for avoiding the recurrence of radial flexion. However, we should pay attention of the radial protrusion of the ulnar head. For avoiding the recurrence of radial deformity or circulatory impairment, gradual correction using Ilizarov external fixation can be indicated, especially in the cases with severe radial deviation or with short forearm. In the mild cases, Bayne Type I or Type II, radius lengthening is accompanied by a soft-tissue distraction or release at the ulnar carpal joint with keeping wrist and forearm motion without producing growth plate damage.

## INTRODUCTION

Radial club hand or radial ray deficiency is a complex congenital abnormality of the radial or pre-axial border of the upper extremity. The term 'radial club hand' is widely used and accepted to describe congenital longitudinal radial ray deficiency = a hand that is radially deviated at the distal forearm in the shape of a golf club [1].

## HISTORY

The first authentic case of congenital absence of the radius was recorded in 1733 by Petit [2]. who described a case of newly-born male infant with bilateral club hand due to total absence of the radius [3].

Since then, particularly throughout the 19th century, several reports of isolated cases based on anatomic dissections have been published [1]. Kato [4] pointed out that Gruber was the first author to make a review of the literature, and referred to 14 cases in 1865. Subsequently, in 1871, Bouvier published an article on 24 cases of the deformity due to complete or partial absence of the radius. Herschel reviewed 32 cases in 1878. Schmid published a statistical study of 48 cases in 1890. Kümmel had found and tabulated 67 cases in 1895. Antonelli reviewed the literature up to 1905 and collected 114 cases, first published in Italian and later translated into German.

Detailed references of all the cases published up until 1923 are included in a 1924 publication by Kato [3]. In this exhaustive review of the literature, Kato had found 250 cases rounded up to the year 1923, and three cases reported in his paper bring the total up to 253 cases. In view of that, Kato's work should perhaps be considered as the first attempt at a comprehensive study of the anatomy, pathology, incidence, clinical presentation, diagnosis, and prognosis of radial dysplasia [1] Skerik and Flatt [4] published an outstanding study on the anatomic variations associated with radial ray deficiency and emphasized the importance of the soft-tissue abnormalities as well as the surgical and functional implications [1].

## EPIDEMIOLOGY AND PREVALENCE

Radial ray deficiency is an uncommon condition, although it is the most common type of longitudinal failure of formation [5]. Radial dysplasia is more common than ulnar dysplasia. The relative prevalence is approximately 2:1 at our institution [6]. Radial ray deficiency is a spectrum of malformations affecting the structures of the radial side of the forearm, including hypoplasia of the bones and joints, muscles and tendons ligaments, nerves, and blood vessels [7]. In the literature, radial club hand deformity is estimated to occur in 1 in 50,000 to 1 in 100,000 live births [8]. The incidence of all radial ray-deficient limbs, including patients with hypoplastic thumbs alone, is approximately 1 in 30,000. The prevalence has been reported to be slightly higher in boys, at 3:2 [6].

## ETIOLOGY

There seems to be a critical period in embryogenesis during which an increased risk of radial ray defects exists. In humans, the upper limb forms between the fourth and eighth postovulatory weeks. The insult, whether environmental or genetic, seems likely to occur in the critical time between weeks 4 and 5 of embryonic development [6].

Although the molecular basis of isolated radial ray deficiency is still unknown, individuals with radial ray deficiency have a high incidence of medical and musculoskeletal anomalies that increases with increasing severity of the deficiency [9].

### VACTERL Association

Radial club hand most commonly includes the heart, the hematopoietic system, and VACTERL (vertebral, anal, cardiopulmonary, tracheoesophageal, renal, and limb; three anomalies must be present to make the diagnosis) [6]. Cases usually occur as sporadic events, although cases in which there is more than one family member affected have been reported [10, 11]. The cause is thought to be a defect in mesodermal development and may be related to defects in the sonic hedgehog signaling pathway [12].

### Holt-Oram Syndrome

In some of the syndromic associations, the genetic lesion has been identified. The association of radial ray deficiency and cardiac anomalies (hand-heart syndrome) was described by Holt and Oram in 1960 [13]. Patients with Holt-Oram syndrome (heart-hand syndrome) have a mutation on chromosome 12 at the location of the *TBX5* gene [14, 15]. Holt-Oram syndrome is an autosomal dominant trait with variable phenotype, and affected families would benefit from genetic counseling [8].

### Fanconi Anemia

Genetic counseling and testing for Fanconi anemia are recommended in the absence of an identifiable syndrome because life-threatening pancytopenia can be treated by bone marrow transplantation [16]. Fanconi anemia is an autosomal recessive disorder characterized by severe hypoplasia or aplasia of the bone marrow with anemia, thrombocytopenia and leukopenia [1]. Despite the identification of numerous Fanconi anemia (*FANC*) genes, the mechanism by these genes encode the proteins that protect a cell from DNA interstrand crosslinks remains unclear [17].

### TAR Syndrome

The TAR (thrombocytopenia-absent radius) syndrome is the association of radial defect and hypomegakaryocytic thrombocytopenia. The cause of TAR syndrome has not yet been identified [6]. The radial ray deficiency in TAR syndrome is almost always characteristic, and the radius is totally absent in affected individuals. The thumb is usually present, although it is hypoplastic. The proximal limb may also be severely foreshortened, giving a phocomelic appearance [6].

### Drugs

Maternal exposure to antiepileptic drugs, particularly valproic acid, has been associated with radial ray deficiency [18]. Other drugs associated with radial ray deficiency include thalidomide, phenobarbital, and aminopterin [6].

## CLINICAL FEATURES

Children with bilateral and severe radial ray deficiency have considerable functional impairment as a result of thumb dysfunction, wrist instability, and short upper extremities. Independent performance of activities of daily living, such as fastening buttons and zippers or accomplishing personal hygiene, is difficult. Objective reproducible hand function testing awaits the development and identification of function tests suitable for children with these malformations [8].

## CLASSIFICATION

Although radial ray deficiency has a wide range of phenotypes from hypoplasia of the thumb to complete absence of the radius and the first ray, Bayne *et al.* classified radial ray deficiency into four types based on the radiographic severity of the radial ray deficiency: Type I, short distal radius (radius is slightly shorter than ulna, distal growth plate is present); Type II, hypoplastic radius (radius is smaller/thinner than ulna, no growth plate is present); Type III, partial absence of the radius (only a small proximal radial segment is present); and Type IV, total absence (radius is completely absent, ulna may be curved) [19].

## TREATMENT - GENERAL PRINCIPLES

The management of radial ray deficiency should start as soon as possible after birth. Treatment should include manipulation and splinting, which will stretch the tight soft tissues and radial structures and allows passive correction of the deformity by aligning the hand and wrist with the ulna. Adequate preoperative soft-tissue stretching is a prerequisite for a successful surgical procedure and should be carried out rigorously until the time of surgery [1].

At the Texas Scottish Rite Hospital in the United States [6], splinting is recommended and it is well tolerated from 0 to 6 months of age. Splints should ideally be above the elbow for increased control because the limb is too small. Serial casting can also be used. After six months of age, splinting is less well tolerated. A stretching program for the parents to follow is then instituted, combined with night splinting if possible. Splinting and stretching are not as effective by two to three years of age. In addition, the 1imb has now a1most doub1ed in size since birth, which makes any subsequent operative treatment easier.

## TREATMENT - SURGERY

Centralization with tendon transfer is a popular method for maintaining the corrected position of radial club hand [19, 20]. The first centralization procedure of the carpus into the distal ulna, an operation that persists until today in modified form, was performed by Sayre in 1894 [21]. On the other hand, various corrections were devised, *e.g*. radialization [22]. after distraction to emphasize the fact that the head of the ulna is positioned under the radial carpal bones and is no longer placed in a slot in the center of the carpus, microvascular epiphysis transfer [23], gradual correction using Ilizarov method [24, 25], for Bayne Type III or Type IV.

To date, there is no established solution for mild radial ray deficiencies and Bayne Type I or Type II. Matsuno *et al.* reported radius lengthening accompanied by a soft-tissue distraction at the ulnar carpal joint and keeping wrist and forearm motion for the mild cases [26]. However, the device was distracted on the ulnar side of the wrist, whereas it is important to distract soft tissues at the radial side for radial ray deficiency. In fact, severe radial deviation of the wrist recurred [26].

Furthermore, we applied it after soft-tissue release at the radial side of the wrist. Correction loss is avoided during growth in our method because the lengthened bone includes the growth plate. In addition, a good range of motion may be also be acquired due to temporary traction of the wrist using an external fixation device without producing growth plate damage [27].

In the present review, we explain our centralization procedure with tendon transfer for Bayne Type III or Type IV, and our bone lengthening of the radius with temporary external fixation of the wrist [27]. for Bayne Type I or Type II.

## CURRENT CONCEPTS IN SURGICAL TECHNIQUE - CENTRALIZATION WITH TENDON TRANSFER

**Fig. (1):**
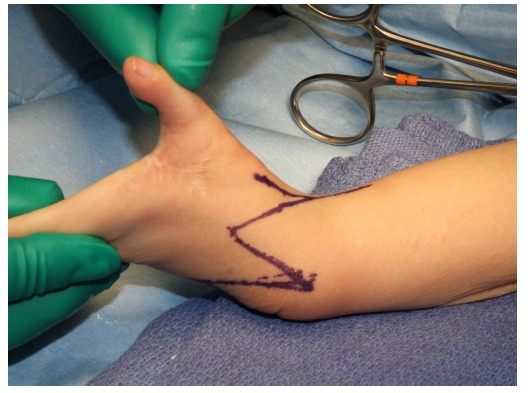
Bilobed flap on the dorsal wrist is designed.

**Fig. (2):**
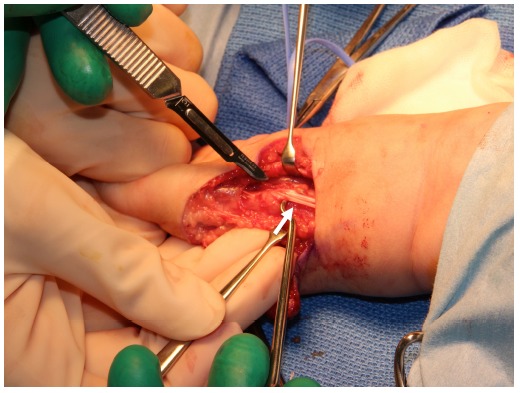
Thick tissue is removed at the radial side for reducing the tension of radial and volar flexion of the wrist with taking care to avoid damage to the superficial branches of the radial nerve.

We design bilobed flap on the dorsal wrist (Fig. **1**). After skin incision, thick tissue is removed at the radial side for reducing the tension of radial and volar flexion of the wrist with care to avoid damage to the superficial branches of the radial nerve (Fig. **2**). Flexor carpi radialis tendon can be detached and is transferred to the dorsal and ulnar side for ulnar deviation and dorsal extension after the correction and fixation of the bones (to be described).

**Fig. (3):**
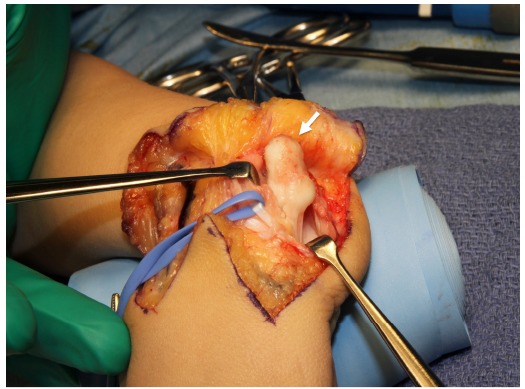
Tissue around the ulnar head is excised and the proximal end of the carpus after extensor retinaculum is cut and extensor carpi ulnaris tendon was retracted to radial side.

Next, we excise tissue around the ulnar head and the proximal end of the carpus after extensor retinaculum is cut and extensor carpi ulnaris tendon was retracted to radial side (Fig. **3**). The proximal end of the carpus is put on the ulnar head. Lunate excision or ulnar shortening can be selected for tension-free correction. Without tension-free correction, the recurrence of radial deformity or circulatory impairment is inevitable.

**Fig. (4):**
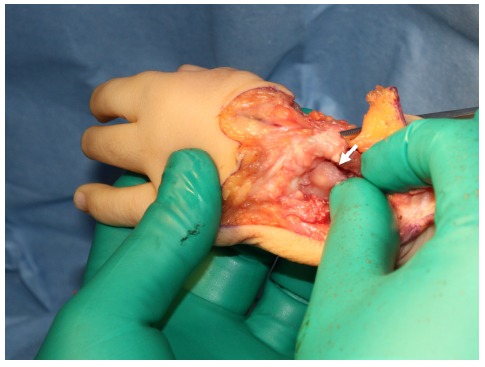
We make sure that the proximal end of the carpus can be on the ulnar head.

When we are certain that carpus is on the ulnar head (Fig. **4**), we insert one or two K-wires from the carpus to metacarpal bone. After the correction between the carpus and the ulnar head, the K-wires are reversely inserted from the metacarpal to the ulna to fix the bones.

Detached flexor carpi radialis tendon is transferred to dorsal and ulnar side on the insertion of flexor carpi ulnaris tendon to help the ulnar deviation and extension.

**Fig. (5):**
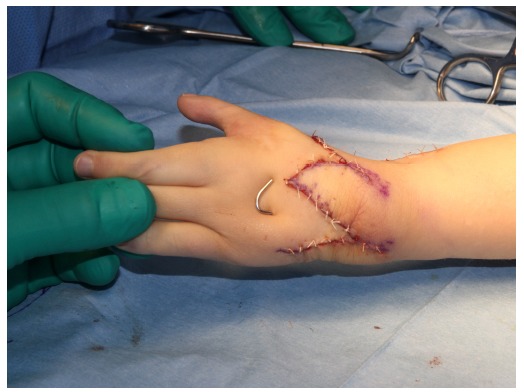
A K-wire is inserted from the metacarpal to the ulna to fix the bones. Bilobed flap is moved to ulnar side and closed with interrupted sutures.

Bilobed flap is moved to the ulnar side and closed with interrupted sutures (Fig. **5**). A long arm splint with bulky soft dressing is applied for four weeks.

The K-wires are removed three months after surgery and active movements of the wrist and digits are permitted. The night brace is also applied to maintain the wrist position one to two years after surgery.

Radialization can be indicated for avoiding the recurrence of radial deviation. However, we should pay attention to the radial protrusion of the ulnar head. To avoid the recurrence of radial deformity or circulatory impairment, gradual correction using Ilizarov external fixation can be indicated, especially in cases with severe radial deviation or with short forearm.

## CURRENT CONCEPTS IN SURGICAL TECHNIQUE - BONE LENGTHENING OF THE RADIUS WITH TEMPORARY EXTERNAL FIXATION OF THE WRIST [27]

**Fig. (6):**
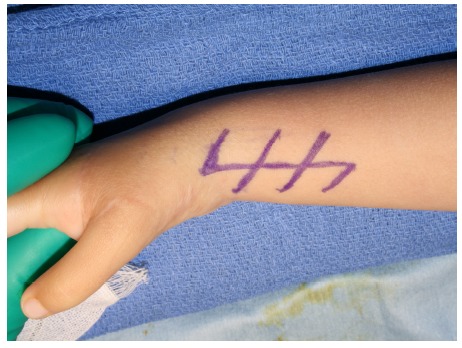
Zigzag incision was made along the radial side of the wrist.

**Fig. (7):**
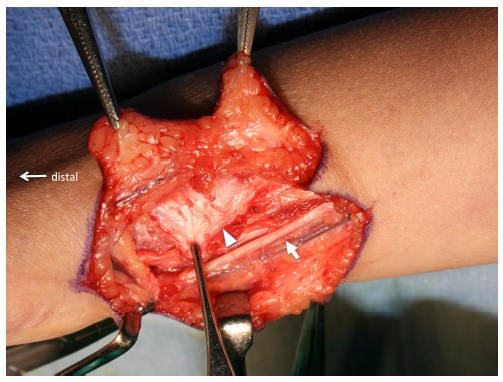
Care was taken to avoid damage to the sensory branches of the radial nerve. The thick fasciae around the brachioradialis and radial wrist extensors were released for improving the range of wrist motion.

A Zigzag incision was made along the radial side of the wrist with care to avoid damage to the sensory branches of the radial nerve (Fig. **6**). The thick fasciae around the brachioradialis and radial wrist extensors were released to improve the range of wrist motion (Fig. **7**).

The distal component including the carpal bones was manually shifted ulnarly after a longitudinal incision was made along the ulnar border of the distal forearm and wrist, on the ulnar side of the sixth dorsal compartment. Care was taken to avoid damage to the sensory branches of the ulnar nerve. We should also be mindful that the distal radius has thick cartilage onto the carpal bones, although it seems to be absent on the X-ray.

The fourth and fifth metacarpal bones and the ulna were fixed with an external fixator (Orthofix M511, Orthofix Orthopedics, Lewisville, TX, USA) in the neutral position of the wrist with a slight traction. Wrist position was confirmed under fluoroscopy.

**Fig. (8):**
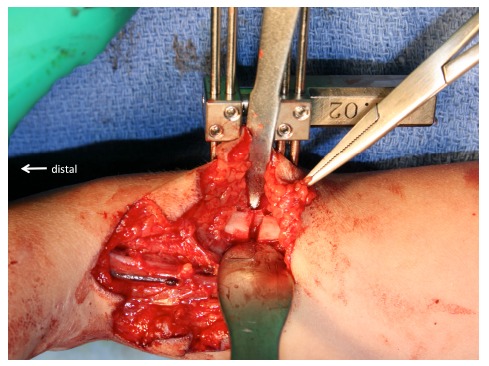
The radius was fixed with an external fixator for bone lengthening after the radial zigzag incision was extended proximally to expose the distal radius.

**Fig. (9):**
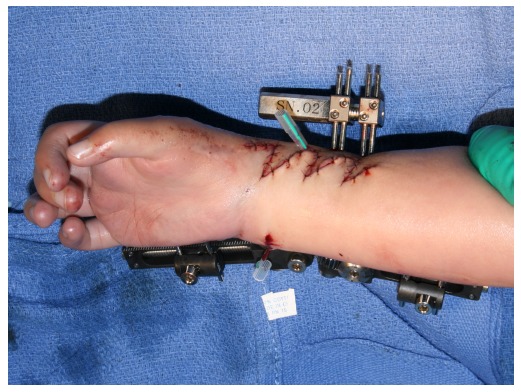
The skin was then closed with interrupted sutures and Z-plasty was applied on the radial side of the wrist.

The radius was fixed with an external fixator for bone lengthening (MES BL2001, ME system, Tokyo, Japan) after the radial zigzag incision was extended proximally to expose the distal radius (Fig. **8**). The skin was then closed with interrupted sutures and Z-plasty was applied to the radial side of the wrist (Fig. **9**). A bulky soft dressing was applied.

Bone lengthening is started five days after the surgery. The lengthening rate is 0.25 to 0.5 mm per day, depending on the condition of bony formation on X-ray, which is evaluated each week. Bone formation is relatively poor in the cases with severe hypoplastic radius. Both external fixators are removed after complete boneformation.

## CONCLUSION

Radial club hand is a complex congenital abnormality of the radial or pre-axial border of the upper extremity. Centralization with tendon transfer is a popular method for maintaining the correct position of radial club hand. In the mild cases, radius lengthening is accompanied by a soft-tissue distraction or release at the ulnar carpal joint with keeping wrist and forearm motion without producing growth plate damage. However, we should pay attention to the recurrence of radial deformity or circulatory impairment with the tension.
